# Genome-wide meta-analysis of cerebral white matter hyperintensities in patients with stroke

**DOI:** 10.1212/WNL.0000000000002263

**Published:** 2016-01-12

**Authors:** Matthew Traylor, Cathy R. Zhang, Poneh Adib-Samii, William J. Devan, Owen E. Parsons, Silvia Lanfranconi, Sarah Gregory, Lisa Cloonan, Guido J. Falcone, Farid Radmanesh, Kaitlin Fitzpatrick, Allison Kanakis, Thomas R. Barrick, Barry Moynihan, Cathryn M. Lewis, Giorgio B. Boncoraglio, Robin Lemmens, Vincent Thijs, Cathie Sudlow, Joanna Wardlaw, Peter M. Rothwell, James F. Meschia, Bradford B. Worrall, Christopher Levi, Steve Bevan, Karen L. Furie, Martin Dichgans, Jonathan Rosand, Hugh S. Markus, Natalia Rost

**Affiliations:** Authors' affiliations are listed at the end of the article.

## Abstract

**Objective:**

For 3,670 stroke patients from the United Kingdom, United States, Australia, Belgium, and Italy, we performed a genome-wide meta-analysis of white matter hyperintensity volumes (WMHV) on data imputed to the 1000 Genomes reference dataset to provide insights into disease mechanisms.

**Methods:**

We first sought to identify genetic associations with white matter hyperintensities in a stroke population, and then examined whether genetic loci previously linked to WMHV in community populations are also associated in stroke patients. Having established that genetic associations are shared between the 2 populations, we performed a meta-analysis testing which associations with WMHV in stroke-free populations are associated overall when combined with stroke populations.

**Results:**

There were no associations at genome-wide significance with WMHV in stroke patients. All previously reported genome-wide significant associations with WMHV in community populations shared direction of effect in stroke patients. In a meta-analysis of the genome-wide significant and suggestive loci (*p* < 5 × 10^−6^) from community populations (15 single nucleotide polymorphisms in total) and from stroke patients, 6 independent loci were associated with WMHV in both populations. Four of these are novel associations at the genome-wide level (rs72934505 [*NBEAL1*], *p* = 2.2 × 10^−8^; rs941898 [*EVL*], *p* = 4.0 × 10^−8^; rs962888 [*C1QL1*], *p* = 1.1 × 10^−8^; rs9515201 [*COL4A2*], *p* = 6.9 × 10^−9^).

**Conclusions:**

Genetic associations with WMHV are shared in otherwise healthy individuals and patients with stroke, indicating common genetic susceptibility in cerebral small vessel disease.

White matter hyperintensities (WMH) on T2-weighted MRI are associated with increasing age and cardiovascular risk factors, particularly hypertension, and are predictive of both stroke and dementia in prospective community populations.^1^ Severe confluent WMH are often found in patients presenting with stroke, and are more common in patients with the small vessel stroke subtype.^2^ Furthermore, in these patients, WMH burden is linked to poor clinical outcomes after stroke.^3,4^ Understanding disease mechanisms that contribute to WMH could lead to advances in prevention, treatment, and rehabilitation of disability related to vascular cognitive impairment, age-related functional decline, and stroke.

Twin and family history studies suggest a significant genetic component to WMH. Heritability estimates range from 55% to 80%,^5–8^ suggesting that a moderate to large proportion of the disease risk can be attributed to genetic effects. The heritability attributed to common single nucleotide polymorphisms (SNPs) has been estimated to be between 13% and 45%.^9^ Previous genome-wide analyses have focused on the genetic influence on WMH in community populations,^10,11^ and a recent meta-analysis identified 8 regions associated with the disease.^12^ One might expect genetic risk factors for WMH in community populations to be similar to those that confer increased risk of WMH in stroke patients. However, the underlying pathology of WMH is heterogeneous, with small punctate lesions being associated with mixed causes, whereas more confluent areas often seen in stroke patients correspond primarily to small vessel disease.^13^ Therefore, it is unclear whether the lesions underlying WMH in the general population are pathologically distinct from the confluent lesions frequently observed in patients with stroke.

In this analysis, we investigated the role of the genetic contribution to WMH volumes (WMHV) in patients with ischemic stroke. We initially performed a genome-wide meta-analysis of WMHV in stroke patients with the aim of identifying novel associations. Second, we determined whether similar genetic factors contributed to WMHV in community populations and stroke patients. Finally, having established shared genetic factors in the 2 datasets, we performed a meta-analysis of the published associations from community populations with our dataset to identify genetic associations that are in common in the 2 populations.

## METHODS

### Study populations.

Ischemic stroke populations were enrolled through hospital-based studies between 1995 and 2013. Characteristics of the study populations are given in table 1; full details are given in the supplementary material on the *Neurology*® Web site at Neurology.org. Patients with cerebral autosomal dominant arteriopathy with subcortical infarcts and leukoencephalopathy or any other suspected monogenic cause of stroke, vasculitis, or any other nonischemic cause of WMH including demyelinating and mitochondrial disorders were excluded from analyses.

**Table 1:**
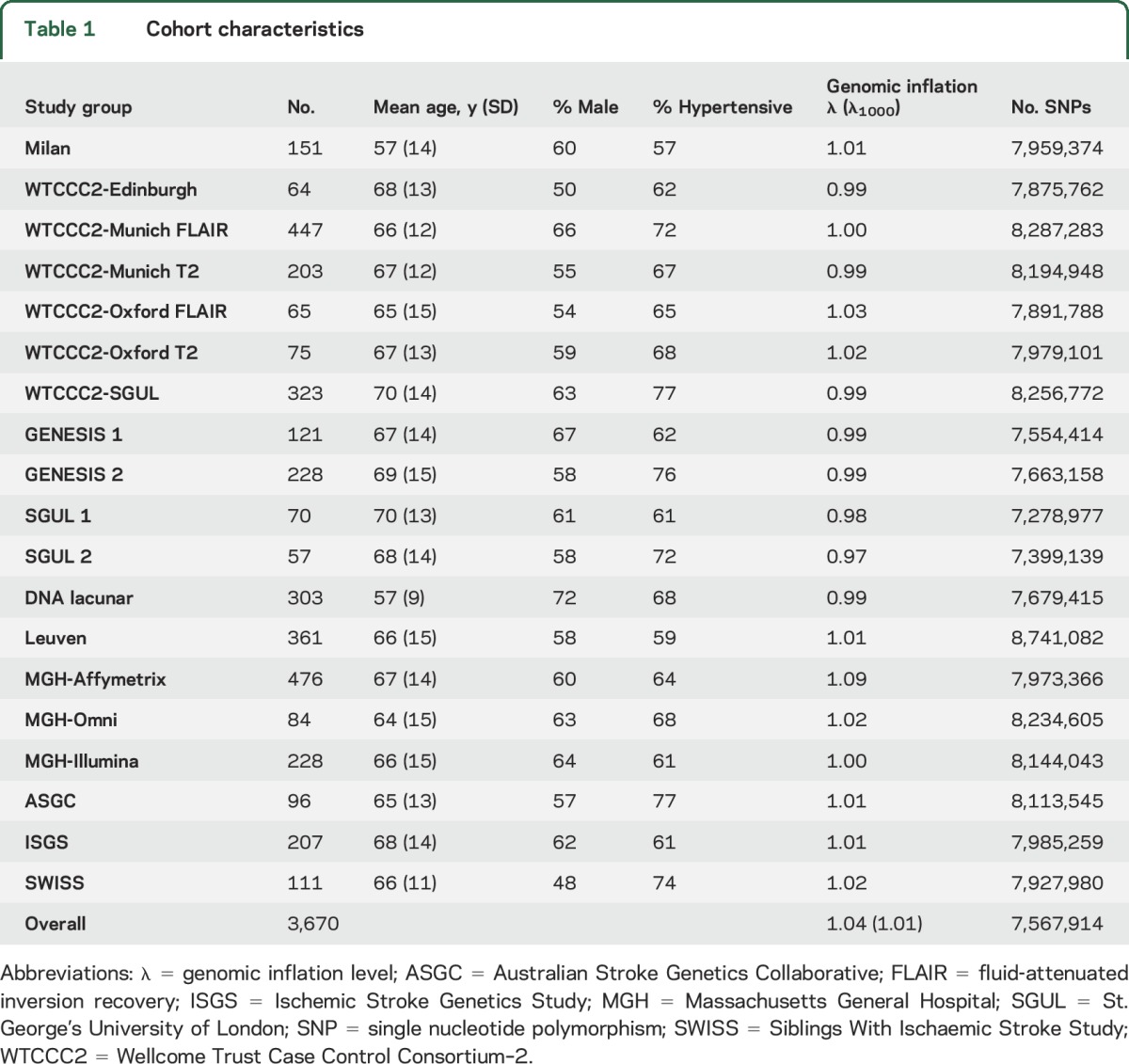
Cohort characteristics

### Standard protocol approvals, registrations, and patient consents.

An institutional review board or regional review board approved the use of human subjects in each of the study populations. All patients gave informed consent.

### Neuroimaging analysis.

MRI scans were acquired as part of routine clinical practice for evaluation of ischemic stroke (table e-1). Fluid-attenuated inversion recovery (FLAIR) sequences were primarily used for WMH volumetric analysis; however, in their absence, T2-weighted sequences were used (Wellcome Trust Case Control Consortium–2 [WTCCC2], Oxford, and WTCCC2, Munich, only). In all scans, to avoid confounding by hyperintense signal due to acute stroke, WMHV was assessed quantitatively in the hemisphere contralateral to the acute infarction. Chronic lacunar infarcts were identified using standard criteria as low signal on T1 or FLAIR images and were excluded from WMHV estimates.^14^ Trained raters blinded to all patient information analyzed anonymized MRI scans. All supratentorial white matter and deep gray matter lesions were included in WMHV with the exception of WMH corresponding to infarcts, both lacunar and territorial.^2^ MRIs with excessive movement artefact, incomplete brain coverage, or bihemispheric infarcts (other than lacunar) were excluded.

To account for interindividual variability in head size, an estimate of total intracranial volume (TICV) was derived using site-specific volumetric methodology, as follows. MRIs from the Massachusetts General Hospital, Ischemic Stroke Genetics Study, and Australian Stroke Genetics Collaborative studies were analyzed in Boston. Scans from the Siblings with Ischaemic Stroke Study were analyzed in the same way at the University of Virginia by the Boston-trained rater. FLAIR sequences were analyzed using an MRIcro semiautomated method as previously described.^2^ Using operator-mediated quality assurances, overlapping regions of interest (ROIs) corresponding to WMH produced the final maps for WMHV calculation. Intracranial area was derived as a validated marker of TICV as the average of 2 midsagittal slices traced using anatomical landmarks on T1 sequences.^15^

The WTCCC2, GENESIS, SGUL, Leuven, and Milan cohorts were analyzed in London using DISPunc semiautomated lesion drawing software.^16^ A seed at the lesion border was first marked manually, and then outlined automatically based on the signal intensity gradient. Each WMH ROI was visually inspected and manually corrected as required. To estimate TICV, T2-weighted and, in their absence, FLAIR sequences were analyzed using an automated segmentation program, SIENAX,^17^ which calculates the total volume of CSF and gray and white matter volumes.

WMHV quantification agreement across the 2 main rating centers was performed for 50 randomly selected scans; agreement was very good (intraclass correlation coefficient 0.95, confidence interval 0.91–0.97, n = 50).

### Phenotype definition.

To calculate the phenotype used in the genetic analysis, WMHV were doubled to obtain a whole brain estimate. This volume was then multiplied by the ratio of TICV (or intracranial area) to the mean TICV (or intracranial area) for the study, thereby correcting for natural differences in head size. The values were natural log transformed and the resulting ln (WMHV) values were entered into a linear regression model including age, sex, and the first 2 ancestry-informative principal components. To ensure the phenotype was normally distributed, the residuals from the model were then *z*-transformed and used as the WMHV phenotype in the genetic analysis.

### Genome-wide genotyping and imputation.

Genotyping of all cohorts was performed on commercially available arrays from Affymetrix (Santa Clara, CA) or Illumina (San Diego, CA) (table e-2). All cohorts performed extensive quality control steps prior to imputation, removing SNPs showing significant departure from Hardy-Weinberg equilibrium, high levels of missingness, or low minor allele frequency. Individuals were removed who did not segregate with Hapmap II European populations based on ancestry informative principal component analysis using EIGENSTRAT or multidimensional scaling in PLINK.^18,19^ Additionally, individuals showing cryptic relatedness or having high levels of missingness or heterozygosity were excluded. All datasets were imputed to 1000 Genomes integrated variant set (March 2012) using IMPUTE v2.^20^

### Genome-wide association analysis of WMHV in stroke patients.

To discover novel associations between WMHV and each autosomal SNP, we performed linear regression of WMHV on genotype dosages using PLINK v1.07.^19^ SNPs with PLINK INFO <0.7 or MAF <0.01 were removed from further analyses. We used genomic inflation to evaluate inflation of test statistics in each study group.^21^ Results across all study groups were combined using a fixed-effects inverse variance weighted method using METAL.^22^ To control for any excess signal that might result from study-wise inflation of *p* values, we performed genomic control correction, multiplying the standard errors from each study by the square root of the genomic inflation factor.^21^ Heterogeneity was assessed using Cochran *q* statistic. Following the meta-analysis, we considered only SNPs present in more than 12 study groups, and with heterogeneity *p* > 0.001, for analysis. We set the significance threshold to *p* < 5 × 10^−8^. We used λ_1000_ to evaluate inflation at the meta-analysis level.^23^ We had 80% power to detect a variant explaining 1.1% of the trait variance (figure e-1).

### Analysis of SNPs associated with WMH in community-based populations.

To determine whether SNPs contributing to WMHV in community populations were associated with WMHV in stroke patients, we evaluated each SNP reported as being associated with WMH in healthy adults in a recent publication,^12^ testing if the SNP was associated with WMHV in ischemic stroke patients. All 17,936 individuals in the previous study were stroke-free and nonoverlapping with the samples studied here. We performed this analysis first for all genome-wide associated loci from the publication, and second for loci reported at *p* < 1 ×10^−5^ in European populations or overall. We set a significance threshold at *p* = 0.0033, Bonferroni correcting for the 15 SNPs analyzed. We had 80% power to detect any associations that explain 0.4% of the trait variance. In addition, we tested whether there was evidence overall that genetic susceptibility factors were shared between the 2 populations. We used a binomial test to evaluate whether an excess of the 8 genome-wide significant SNPs shared direction of effect in community populations and stroke patients, and then extended this to the 15 genome-wide significant loci and loci reported at *p* < 1 × 10^−5^ in European populations or overall.

### Meta-analysis of stroke samples and published population-based samples.

Having established that genetic factors were shared between community populations and stroke patients, we evaluated the overall evidence that each of the 15 previously reported SNPs (8 genome-wide significant, 7 suggestive) were associated with WMHV in both populations. We combined *p* values from the 2 sources using Stouffer *z*-score weighted method with equal weights, classifying SNPs with *p* < 0.05 in both populations and reaching *p* < 5 × 10^−8^ overall as significantly associated with WMH in both populations. We were not able to perform the reciprocal analysis, testing if suggestive associations with WMH in stroke patients were associated with WMH in stroke-free individuals, due to restrictions on access to the required summary level data. We then evaluated novel genome-wide associations in available databases to test for evidence that affects regulation of genes (RegulomeDB)^24^ or directly affects gene expression (GTEx).^25^

## RESULTS

### Study populations.

Clinical characteristics of all participating cohorts are given in table 1. In total, 3,670 individuals of European ancestry were included in the 19 study groups.

### Genome-wide association analysis of WMHV in stroke patients.

**Figure 1:**
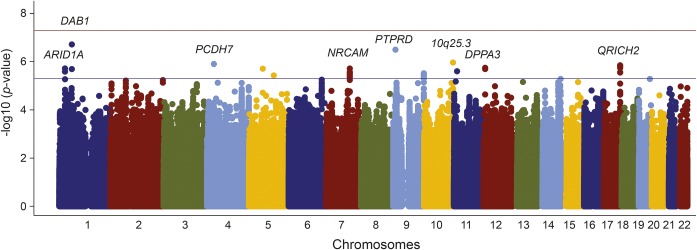
Association of genome-wide single nucleotide polymorphisms with white matter hyperintensity volume in ischemic stroke patients by genomic position

With the exception of one study group, genomic inflation was well-controlled (λ ≤ 1.03, table 1). Following quality control procedures, 7,567,914 autosomal SNPs remained for analysis. Genomic inflation was well-controlled at the meta-analysis level (λ = 1.04, λ_1000_ = 1.01; figure e-2). No SNP reached the significance level (figure 1), although a number of loci reached *p* < 5 × 10^−6^. These are detailed in table e-3, and regional plots of these loci are provided in figure e-3. All odds ratios reported are per 1 SD change in normally distributed WMHV after accounting for age, sex, and ancestry-informative principal components.

### Analysis of SNPs associated with WMH in community-based populations.

Eight independent SNPs have been associated with WMH in community populations.^12^ We evaluated each of these in our dataset of stroke patients. The direction of effect of all 8 associations was consistent with the direction in our study. This alone is unlikely to be due to chance (*p* = 7.8 × 10^−3^ from binomial test). For specific SNPs, no genome-wide associations from community populations reached our significance threshold, although all had *p* ≤ 0.24 for association with WMH in stroke patients, and 3 loci reached a nominal significance level (*p* < 0.05) in stroke patients (rs7214628 [*TRIM65*], *p* = 0.015; rs78857879 [*EFEMP1*], *p* = 0.0056; rs2984613 [*PMF1-BGLAP*], *p* = 0.017).

Additionally, 10 loci were reported as suggestively significant in the same recent publication,^12^ with *p* < 1 × 10^−5^ in Europeans or overall. Three of these were rare (MAF ≤ 0.02), and were not imputed with enough accuracy to be analyzed in our dataset (rs186314186, rs150695384, rs117126031). We evaluated each of the 7 remaining associations in our population. Of these, 4 passed our significance threshold (table 2). One locus was nonsignificant and in the opposite direction in our study (rs2883428, *p* = 0.17). In total, 14 of the 15 genome-wide and suggestively significant loci shared direction between community individuals and stroke patients (*p* = 9.8 × 10^−4^ from binomial test).

**Table 2:**
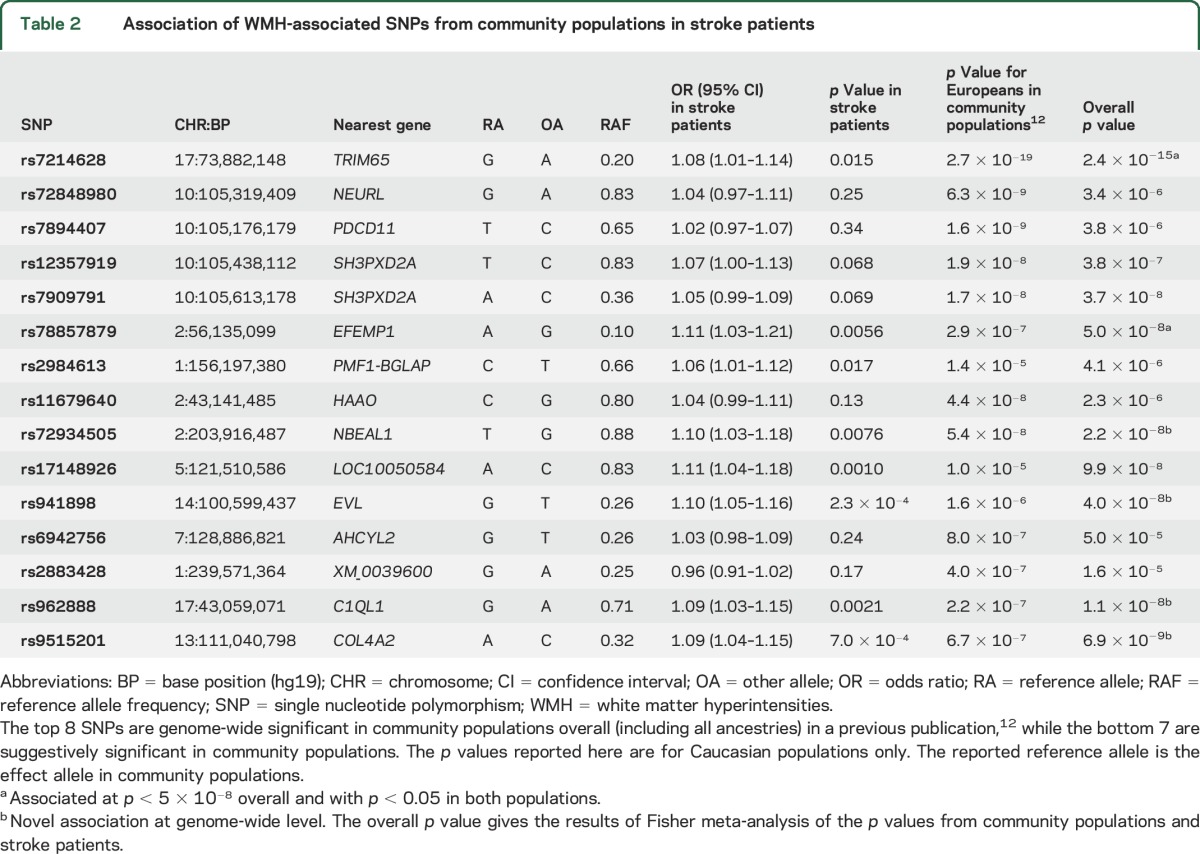
Association of WMH-associated SNPs from community populations in stroke patients

In addition, we searched for other publications describing associations with any of the SNPs or genes studied using the following search terms in PubMed: (SNP or gene) and (white matter or leukoaraiosis or small vessel disease). No relevant publications were identified.

### Meta-analysis of stroke samples and published population-based samples.

When combining our results in stroke patients with the 15 previously reported associations using Stouffer *z*-score meta-analysis, 6 associations reached genome-wide significance overall and had *p* < 0.05 in both studies (table 2). Four of these are novel associations at genome-wide significance (rs72934505 [*NBEAL1*], *p* = 2.2 × 10^−8^; rs941898 [*EVL*], *p* = 4.0 × 10^−8^; rs962888 [*C1QL1*], *p* = 1.1 × 10^−8^; rs9515201 [*COL4A2*], *p* = 6.9 × 10^−9^), all of which showed good consistency across the 19 cohorts (figure e-4). The same 6 associations reached genome-wide significance using an alternative meta-analysis approach (Fisher method). The association with *COL4A2* (rs9515201) is in strong LD (r^2^ > 0.8) with SNPs previously reported to be associated with cerebral small vessel disease, and is therefore likely to represent the same locus.^26^

For each of these 4 novel associations, we queried the RegulomeDB database and GTEx portal for evidence that the SNPs affect DNA binding or expression of any mRNA molecule (figure e-5).^24,25^ rs962888 lies 25 Kb downstream from *C1QL1*; however, interrogation of GTEx portal showed that the common allele (G, risk allele) of the SNP decreases expression of elongation factor tu GTP binding domain containing 2 (EFTUD2) in tibial arteries, 100 kb away (*p* = 5.3 × 10^−6^). Data from RegulomeDB support this observation, as the SNP overlies DNase-seq, FAIRE-seq, and CHIP-seq peaks in numerous tissues from ENCODE.^27^ Similarly, the common allele (T, risk allele) of rs72934505 increases expression of the nearby gene *NBEAL1* in tibial arteries in GTEx (*p* = 2.5 × 10^−11^), and also decreases expression of islet cell autoantigen 1.69 kDa-Like (*ICA1L*) in the thyroid (*p* = 6.6 × 10^−6^), 200 kb away. No significant eQTLs were identified for rs941898 or rs9515201, but both overlap numerous CHIP-seq and DNAse-seq peaks from ENCODE, indicating they may have a regulatory function.

## DISCUSSION

We report the first phase of a collaborative genome-wide meta-analysis of WMHV in stroke patients. We did not identify any associations with WMHV in ischemic stroke patients at the genome-wide significance level. The most likely explanation for this is lack of power. We had 80% power to identify a variant explaining 1.1% of the trait variance (supplementary material), suggesting that it is unlikely that any common variants explain more than this proportion of the variance of WMH in stroke patients. However, we cannot rule out the existence of rare variants conferring a considerable proportion of disease risk.

We found strong evidence that many of the same genome-wide associations with WMHV in healthy individuals influence WMHV in stroke patients. All genome-wide significant associations with WMHV shared direction of effect in our study and 3 reached a nominal significance threshold. More convincing is that of the 7 suggestive associations reported with WMH in healthy individuals, 4 were significantly associated with WMH in stroke patients. A meta-analysis of these SNPs in 21,606 subjects suggests that 4 of these loci are linked to WMH in community populations and stroke patients at genome-wide significance. Two of these associations influence expression of nearby gene products (*NBEAL1/ICA1L* [rs72934505] and *EFTUD2* [rs962888]). A genome-wide significant association with rs9515201, located in an intron of *COL4A2*, which encodes collagen 4 subunit 2, was also identified. This association is particularly interesting as rare mutations in *COL4A2* and the closely related *COL4A1* protein lead to small vessel disease and hemorrhagic stroke,^28–30^ and common variants in close LD with this SNP (*r*^*2*^ > 0.8) have been linked to sporadic small vessel disease.^26^

The observation that genetic risk factors for WMH in community populations also influence WMH in stroke patients has implications. It suggests that the white matter changes seen on the brain MRI scans of otherwise healthy elderly reflect a similar disease process as the more severe forms that underlie cerebral small vessel disease in patients with stroke. Previous studies have indicated heterogeneity in WMH pathology: our results do not preclude this possibility, but suggest that many of the same genetic factors contribute to both pathologies.

Our study has several strengths. Protocols were uniformly employed across analyses, including imputation to the same reference build across all study groups, using the same software. Similarly, analyses were performed using the same software on the same phenotype, derived in the same way. We performed volumetric analysis of all MRI scans to quantify WMHV, which has strengths over rating scales, which are known to have ceiling effects.^14^ Inter-rater agreement between the 2 coordinating centers was shown to be good. WMHV was quantified using semiautomated volumetric protocols validated for use in patients with stroke and clinical grade MRI scans.

Our study also has limitations. Large-scale collaborative GWAS such as that undertaken here necessarily combine studies with some degree of phenotypic variability. Differences in environmental exposures, possibly resulting in epigenetic modifications, may contribute to such variability, which could alter the results. We identified 4 novel associations at genome-wide significance when combined with previous publications. However, we have not provided replication of these findings and therefore further evidence will be necessary to verify these associations with WMHV. MRI used in the analyses were drawn from a number of centers, with varying image quality. Therefore, to minimize bias arising from differing image quality, we quantified WMHV per study group and meta-analyzed the results. This approach may limit our ability to detect associations with low frequency variants due to small sample sizes in some study groups. The majority of MRI scans used were from FLAIR sequences. However, where these were unavailable, we used T2-weighted images, which are less sensitive to white matter changes. Such differences in sensitivity may affect quantification of WMHV across study groups, although future studies that involve centralized volumetric MRI analysis pipelines, such as those currently in development, may account for this variability. In this analysis, we considered all subtypes of stroke together as we were underpowered to investigate subtype-specific influences on WMH. It is possible that causes of WMH may differ by stroke subtype, but larger studies with sufficient power will be required before this issue can be addressed adequately. Similarly, it has been hypothesized that periventricular and deep WMH might have distinct underlying pathophysiology. In this analysis, we considered total WMHV, rather than treating these regions separately; our lesion volume analysis did not differentiate into these 2 regions. Further analyses should address this area.

We have shown that the age-related white matter changes seen in otherwise healthy populations share genetic susceptibility with the extensive lesions that underlie cerebral small vessel disease. We report 6 independent loci that are associated with WMHV in healthy individuals as well as stroke patients, 4 of which are novel associations at the genome-wide level. Our results suggest that a full genome-wide meta-analysis of available cohorts of WMH in ischemic stroke patients and community populations is likely to uncover further associations.

## Supplementary Material

Data Supplement

Coinvestigators

## AUTHOR AFFILIATIONS

From the Department of Clinical Neurosciences (M.T., H.S.M.) and the Autism Research Centre (O.E.P.), University of Cambridge, UK; the J. Philip Kistler Stroke Research Center (C.R.Z., W.J.D., L.C., G.J.F., K.F., A.K., N.R.) and the Center for Human Genetic Research (W.J.D., G.J.F., F.R., J.R., N.R.), Department of Neurology, Massachusetts General Hospital, Boston; the Neurosciences Research Centre (P.A.-S., S.G., T.R.B., B.M.), St George's, University of London, UK; the Neurology Unit (S.L., J.R.), IRCCS Ca' Granda Ospedale Maggiore Policlinico, Milan, Italy; Program in Medical and Population Genetics (F.R.), Broad Institute, Cambridge, MA; the Department of Medical & Molecular Genetics (C.M.L.) and the Social, Genetic and Developmental Psychiatry Centre, Institute of Psychiatry (C.M.L.), King's College London, UK; the Department of Cerebrovascular Diseases (G.B.B.), Fondazione IRCCS Istituto Neurologico “Carlo Besta,” Milan, Italy; KU Leuven, University of Leuven, the Department of Neurosciences, Experimental Neurology and Leuven Research Institute for Neuroscience and Disease (R.L., V.T.); University Hospitals Leuven, the Department of Neurology (R.L., V.T.); VIB, Vesalius Research Center, Laboratory of Neurobiology (R.L., V.T.), Leuven, Belgium; Austin Health and Melbourne Brain Center (V.T.), Florey Institute of Neuroscience and Mental Health, University of Melbourne, Heidelberg, Victoria, Australia; the Division of Clinical Neurosciences, Neuroimaging Sciences and Institute of Genetics and Molecular Medicine (C.S.), and Neuroimaging Sciences, Centre for Clinical Brain Sciences (J.W.), University of Edinburgh, UK; the Stroke Prevention Research Unit (P.M.R.), Nuffield Department of Neuroscience, University of Oxford, UK; the Department of Neurology (J.F.M.), Mayo Clinic, Jacksonville, FL; the Departments of Neurology and Public Health Sciences (B.B.W.), University of Virginia, Charlottesville; the Centre for Clinical Epidemiology and Biostatistics (C.L.), Hunter Medical Research Institute and School of Medicine and Public Health, University of Newcastle, NSW, Australia; School of Life Science (S.B.), University of Lincoln, Lincoln, UK; the Department of Neurology (K.L.F.), Rhode Island Hospital, Alpert Medical School of Brown University, Providence; the Institute for Stroke and Dementia Research (M.D.), Klinikum der Universität München, Ludwig-Maximilians-University Munich; and the Munich Cluster for Systems Neurology (SyNergy) (M.D.), Munich, Germany.

## GLOSSARY

FLAIR: fluid-attenuated inversion recovery
ROI: region of interest
SNP: single nucleotide polymorphism
TICV: total intracranial volume
WMH: white matter hyperintensities
WMHV: white matter hyperintensity volume
WTCCC2: Wellcome Trust Case Control Consortium–2
