# Supplementary material for: Genome-wide meta-analysis of cerebral white matter hyperintensities in patients with stroke
Source: Neurology. 2016 Jan 12;86(2):146–53. doi: 10.1212/WNL.0000000000002263 (PMC4731688; doi:10.1212/WNL.0000000000002263)
Supplement: Data Supplement [file supp_86_2_146__index.html]

Data Supplement 

# Genome-wide meta-analysis of cerebral white matter hyperintensities in patients with stroke

## Data Supplement

Three tables, five figures, and supplemental data; seven PDF files.

**Neurology® data supplements are not copyedited before publication. Published editorials and translations have been copyedited.  
 © 2016 American Academy of Neurology.  
  
 Files in this Data Supplement:**

- Tables e-1 to e-3 - PDF file
- Figure e-1 - PDF file
- Figure e-2 - PDF file
- Figure e-3 - PDF file
- Figure e-4 - PDF file
- Figure e-5 - PDF file
- Supplemental data - PDF file
