# Supplementary material for: Genome-wide meta-analysis of cerebral white matter hyperintensities in patients with stroke
Source: Neurology. 2016 Jan 12;86(2):146–53. doi: 10.1212/WNL.0000000000002263 (PMC4731688; doi:10.1212/WNL.0000000000002263)
Supplement: Data Supplement [file supp_WNL.0000000000002263_e-tables.pdf]

Table e-1 - Magnetic resonance imaging (MRI) sequences and MRI models for cohorts in WMH GWAS.

Centre	WMHV Sequence	TICV/ICA sequence	MRI scanner
WTCCC2-SGUL	Axial FLAIR	Axial T2	1.5T Philips, 1.5T GE Signa LX
WTCCC2-Oxford FLAIR	Coronal FLAIR	Axial T2	1.5T GE Medical Signa, 1.5T Philips
WTCCC2-Oxford T2	Axial T2-weighted	Axial T2	1.5T GE Medical Signa, 1.5T Philips
WTCCC2-Edinburgh	Axial FLAIR	Axial FLAIR	1.5T GE Medical Signa, 1.5T Philips
WTCCC2-Munich FLAIR	Axial FLAIR	Axial FLAIR	1.5T Siemens Magnetom, 1T Siemens, 1.5T GE Medical Signa
WTCCC2-Munich T2	Axial T2-weighted	Axial T2	1.5T Siemens Magnetom, 3T & 1.5T GE Medical Signa, 1T Siemens
Milan	Axial FLAIR and Coronal FLAIR	Axial T2	1.5T Siemens, 0.5T Philips
ISGS	Axial FLAIR	Sagittal T1	1.5T GE Medical Signa
MGH Affymetrix	Axial FLAIR	Sagittal T1	1.5T GE Medical Signa
MGH Illumina	Axial FLAIR	Sagittal T1	1.5T GE Medical Signa
MGH Omni	Axial FLAIR	Sagittal T1	1.5T GE Medical Signa
SWISS	Axial FLAIR	Sagittal T1	1.5T GE Medical Signa
ASGC	Axial FLAIR	Sagittal T1	1.5T Siemens Magnetom Avanto
DNA Lacunar	Axial FLAIR	Axial T2	Multiple Scanners
GENESIS (1 & 2)	Axial FLAIR	Axial T2	1.5T Philips, 1.5T GE Signa LX
SGUL (1 & 2)	Axial FLAIR	Axial T2	1.5T Philips, 1.5T GE Signa LX
Leuven	Axial FLAIR	Axial T2	1.5T GE Medical Signa

SGUL, St. George’s University of London; MGH, Massachusetts General Hospital; ASGC, Australian Stroke Genetics Collaborative; ISGS, Ischemic Stroke Genetics Study; SWISS, Siblings With Ischaemic Stroke Study;

Table e-2 - Genotyping and imputation in all centres

Centre	Genotyping	Imputation
WTCCC2-SGUL	Illumina Human660W-Quad	1000 Genomes integrated variant set (March 2012)
WTCCC2-Oxford FLAIR	Illumina Human660W-Quad	1000 Genomes integrated variant set (March 2012)
WTCCC2-Oxford T2	Illumina Human660W-Quad	1000 Genomes integrated variant set (March 2012)
WTCCC2-Edinburgh	Illumina Human660W-Quad	1000 Genomes integrated variant set (March 2012)
WTCCC2-Munich FLAIR	Illumina Human660W-Quad	1000 Genomes integrated variant set (March 2012)
WTCCC2-Munich T2	Illumina Human660W-Quad	1000 Genomes integrated variant set (March 2012)
Milan	Illumina Human610-Quad v1_B, Human660W-Quad v1_A	1000 Genomes integrated variant set (March 2012)
ISGS	Illumina 660W-Quad	1000 Genomes integrated variant set (March 2012)
MGH Affymetrix	Affymetrix 6.0	1000 Genomes integrated variant set (March 2012)
MGH Illumina	Illumina Human610-Quad	1000 Genomes integrated variant set (March 2012)
MGH Omni	Illumina OmniExpress	1000 Genomes integrated variant set (March 2012)
SWISS	Illumina 660W-Quad	1000 Genomes integrated variant set (March 2012)
ASGC	Illumina Human610-Quad	1000 Genomes integrated variant set (March 2012)
DNA Lacunar	Illumina HumanExomeCore	1000 Genomes integrated variant set (March 2012)
GENESIS (1 & 2)	Illumina HumanExomeCore	1000 Genomes integrated variant set (March 2012)
SGUL (1 & 2)	Illumina HumanExomeCore	1000 Genomes integrated variant set (March 2012)
Leuven	Illumina Omni 5M	1000 Genomes integrated variant set (March 2012)

SGUL, St. George's University of London; MGH, Massachusetts General Hospital; ASGC, Australian Stroke Genetics Collaborative; ISGS, Ischemic Stroke Genetics Study; SWISS, Siblings With Ischaemic Stroke Study;

Table e-3 – Top eight loci suggestively associated WMH in patients with ischaemic stroke

SNP	CHR:BP	Gene	RA	OA	RAF	OR (95% CI)	P-value
rs17579352	1:58350805	*DAB1*	C	T	0.95	1.40 (1.23–1.59)	$1.9x10^{-7}$
rs1535459	9:10550204	*PTPRD*	C	G	0.98	1.87 (1.47–2.38)	$3.2x10^{-7}$
rs7082237	10:131889797	*N/A*	C	T	0.88	1.20 (1.12–1.29)	$1.1x10^{-6}$
rs3733655	4:31146018	*PCDH7*	A	G	0.64	1.13 (1.07–1.18)	$1.3x10^{-6}$
rs346785	17:74283769	*QRICH2*	T	C	0.61	1.12 (1.07–1.18)	$1.4x10^{-6}$
rs73267033	12:7864476	*DPPA3*	A	G	0.96	1.45 (1.25–1.69)	$1.8x10^{-6}$
rs11247594	1:27067417	*ARID1A*	G	A	0.79	1.15 (1.09–1.22)	$1.9x10^{-6}$
rs11974528	7:107832411	*NRCAM*	C	T	0.98	1.53 (1.29–1.83)	$1.9x10^{-6}$

CHR, chromosome; BP, base position; RA, risk allele; OA, other allele; RAF, risk allele frequency; OR, odds ratio; CI, confidence interval. Heterogeneity p-value is Cochran's q statistic.
