# Supplementary material for: Genome-wide meta-analysis of cerebral white matter hyperintensities in patients with stroke
Source: Neurology. 2016 Jan 12;86(2):146–53. doi: 10.1212/WNL.0000000000002263 (PMC4731688; doi:10.1212/WNL.0000000000002263)
Supplement: Data Supplement [file supp_WNL.0000000000002263_Figure_e-1.pdf]

Figure e-1 – Power to detect a causal locus explaining a given proportion of trait variance for a quantitative trait with sample size 3,670

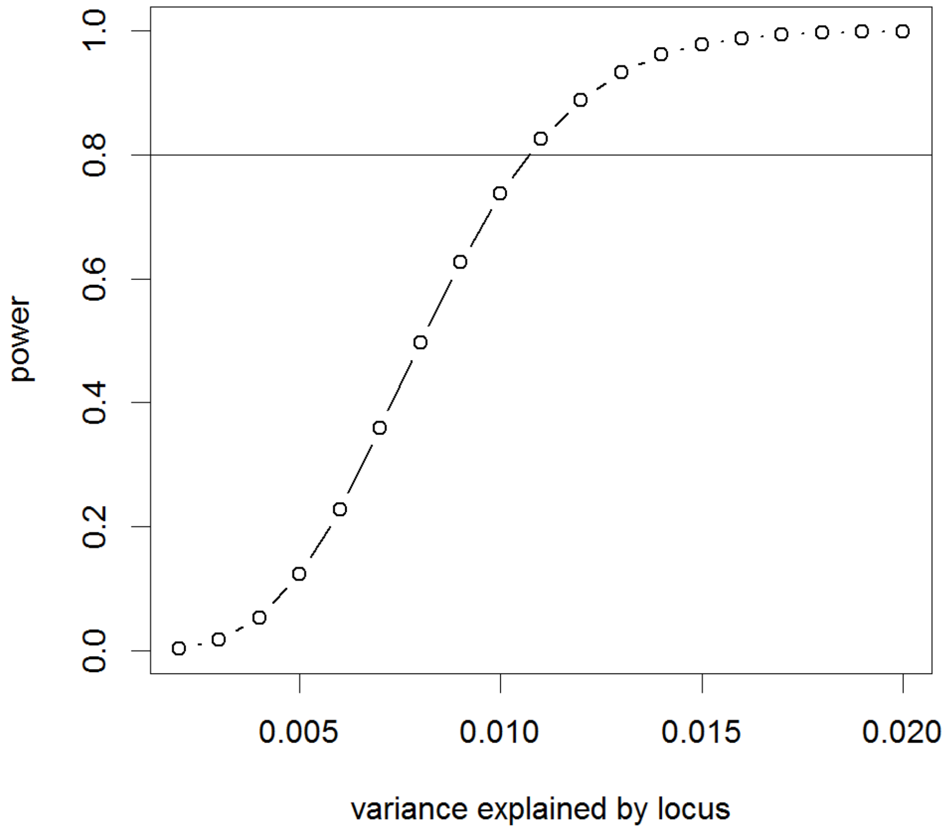

Horizontal line at 80% power.
