# Supplementary figures and images for: Genome-wide meta-analysis of cerebral white matter hyperintensities in patients with stroke
Source: Neurology. 2016 Jan 12;86(2):146–53. doi: 10.1212/WNL.0000000000002263 (PMC4731688; doi:10.1212/WNL.0000000000002263)

**Figure e-2** – QQ-plot of expected against observed test statistics, with 95% confidence intervals

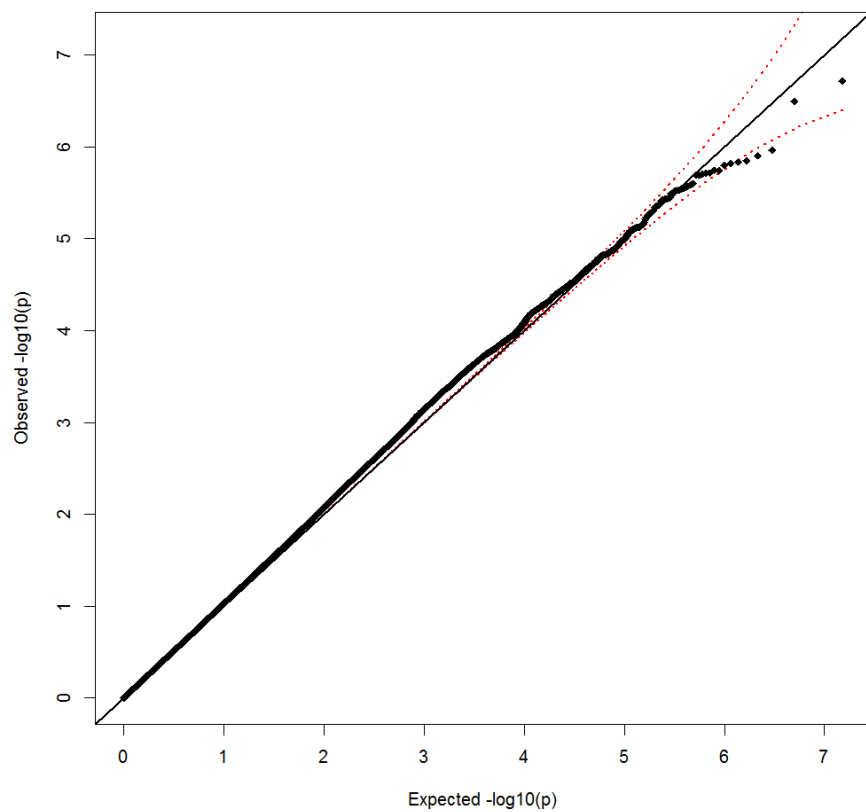

Supplement: Data Supplement [file supp_WNL.0000000000002263_Figure_e-2.pdf]
