# Supplementary material for: Genome-wide meta-analysis of cerebral white matter hyperintensities in patients with stroke
Source: Neurology. 2016 Jan 12;86(2):146–53. doi: 10.1212/WNL.0000000000002263 (PMC4731688; doi:10.1212/WNL.0000000000002263)
Supplement: Data Supplement [file supp_WNL.0000000000002263_Figure_e-3.pdf]

Figures e-3 – Regional association plots for loci suggestively associated with WMH in stroke patients

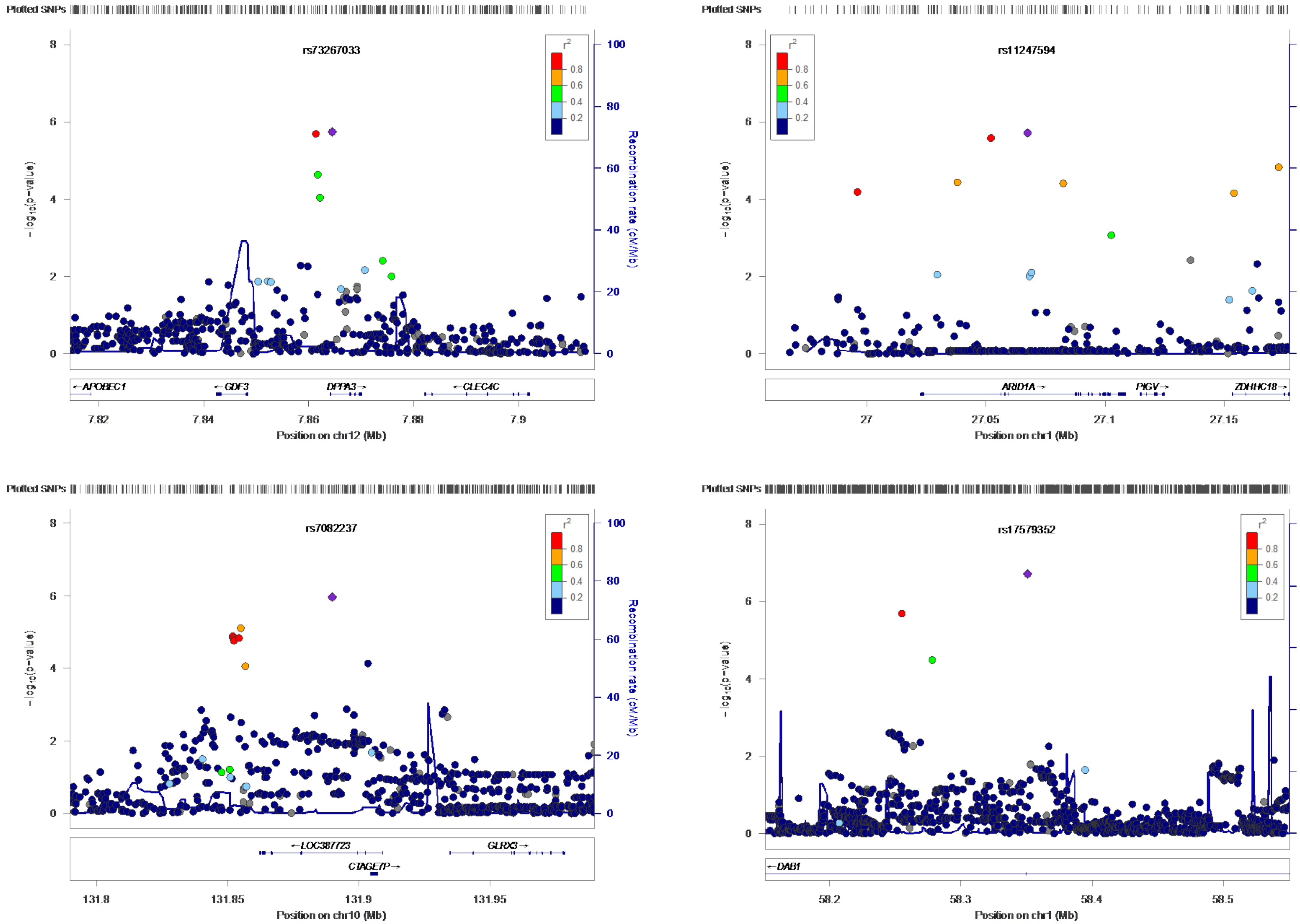

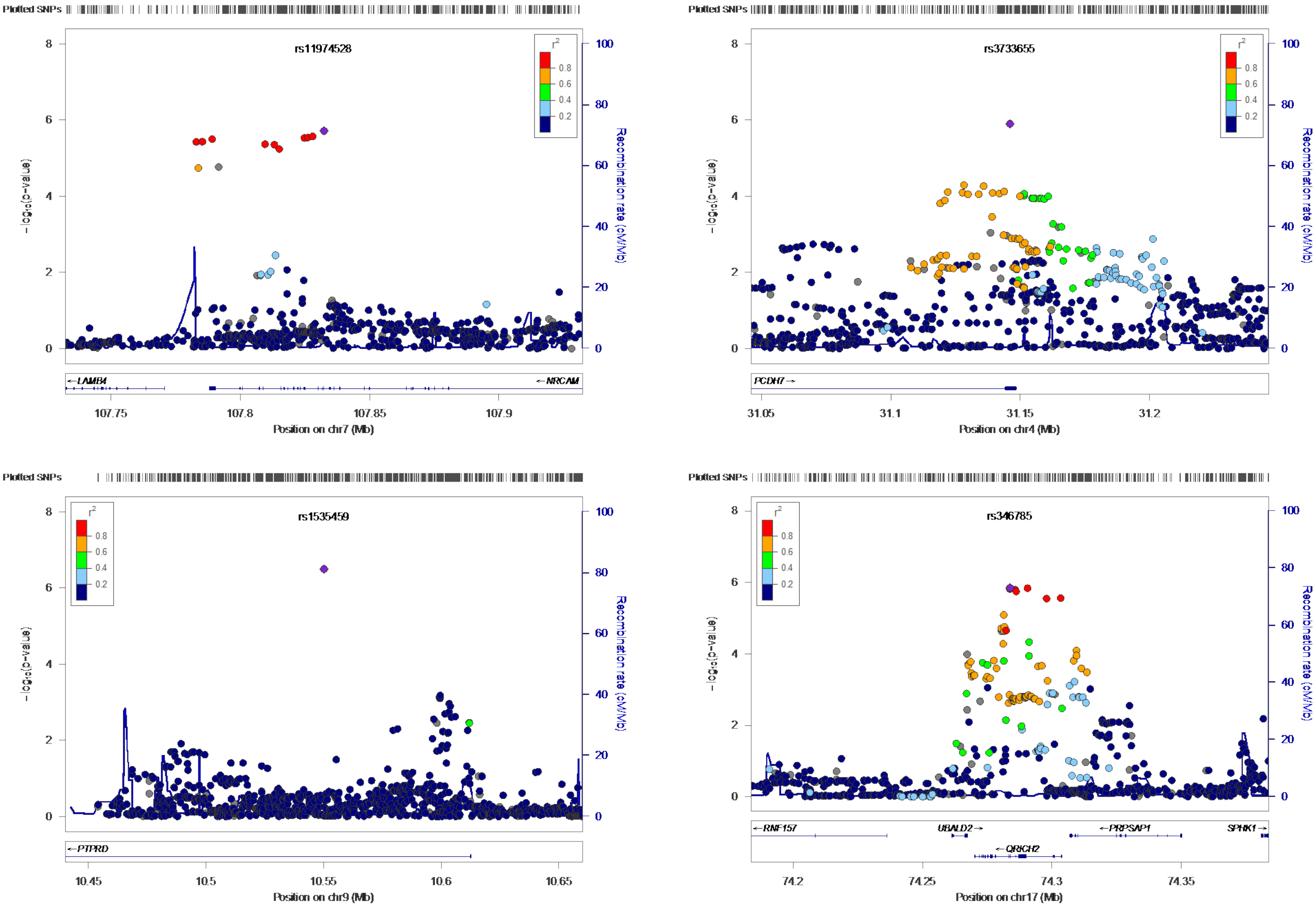
Plotted SNPs
rs11974528
LAMB4
NRCAM
Position on chr7 (Mb)
rs3733655
PCDH7
Position on chr4 (Mb)
rs1535459
PTPRD
Position on chr9 (Mb)
rs346785
RNF157
UBALD2
QRICH2
PRPSAP1
SPHK1
Position on chr17 (Mb)
$-\log_{10}$(p-value)
Recombination rate (cM/Mb)
r^2
