# Supplementary material for: Genome-wide meta-analysis of cerebral white matter hyperintensities in patients with stroke
Source: Neurology. 2016 Jan 12;86(2):146–53. doi: 10.1212/WNL.0000000000002263 (PMC4731688; doi:10.1212/WNL.0000000000002263)

Figure e-4 - Forest Plots of novel loci associated with WMH in stroke patients and in community populations

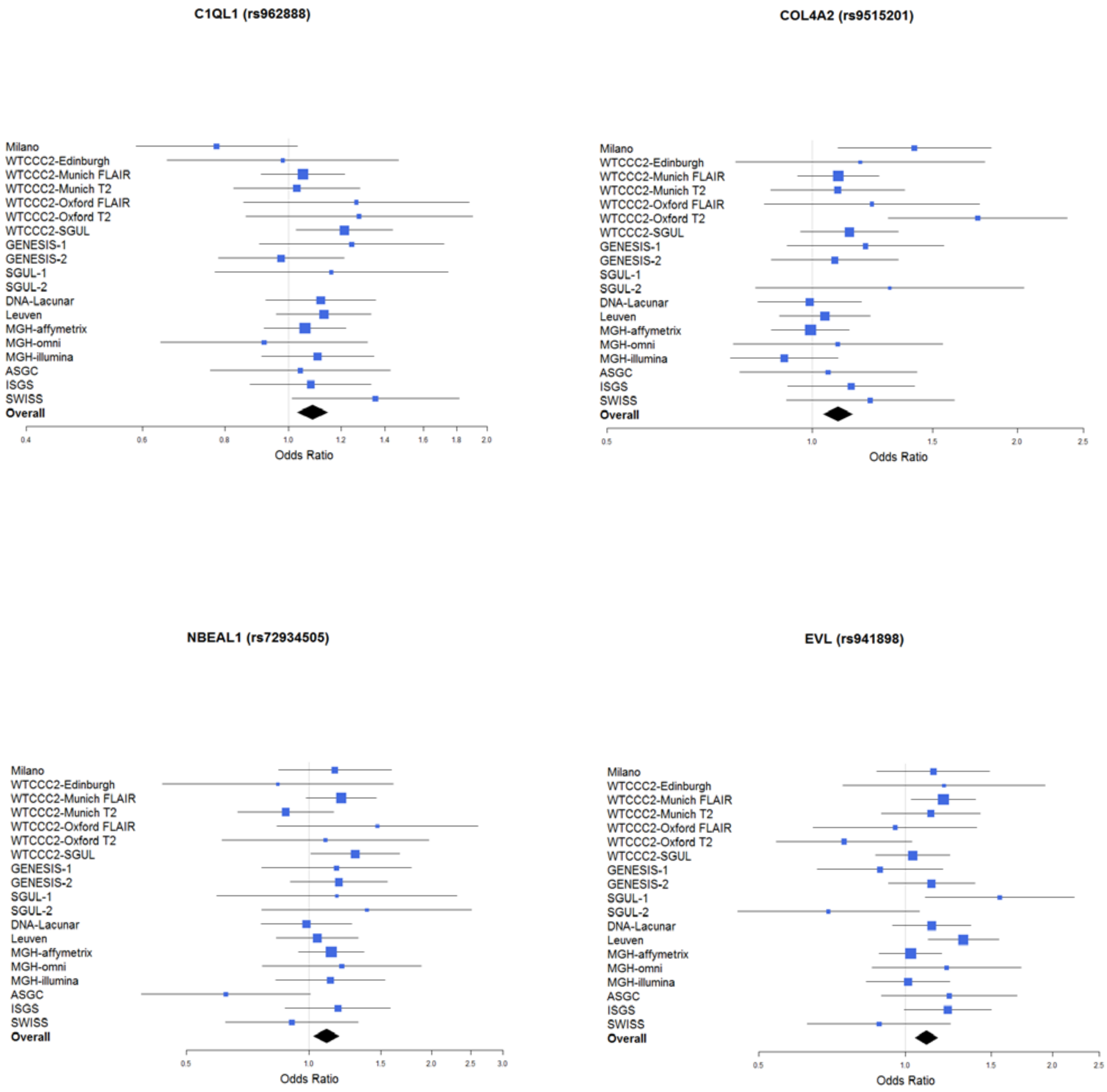

Supplement: Data Supplement [file supp_WNL.0000000000002263_Figure_e-4.pdf]
