# Supplementary material for: Genome-wide meta-analysis of cerebral white matter hyperintensities in patients with stroke
Source: Neurology. 2016 Jan 12;86(2):146–53. doi: 10.1212/WNL.0000000000002263 (PMC4731688; doi:10.1212/WNL.0000000000002263)
Supplement: Data Supplement [file supp_WNL.0000000000002263_Figure_e-5.pdf]

Figure e-5 – Evidence that rs72934505 and rs962888 influence expression of associated mRNA molecules

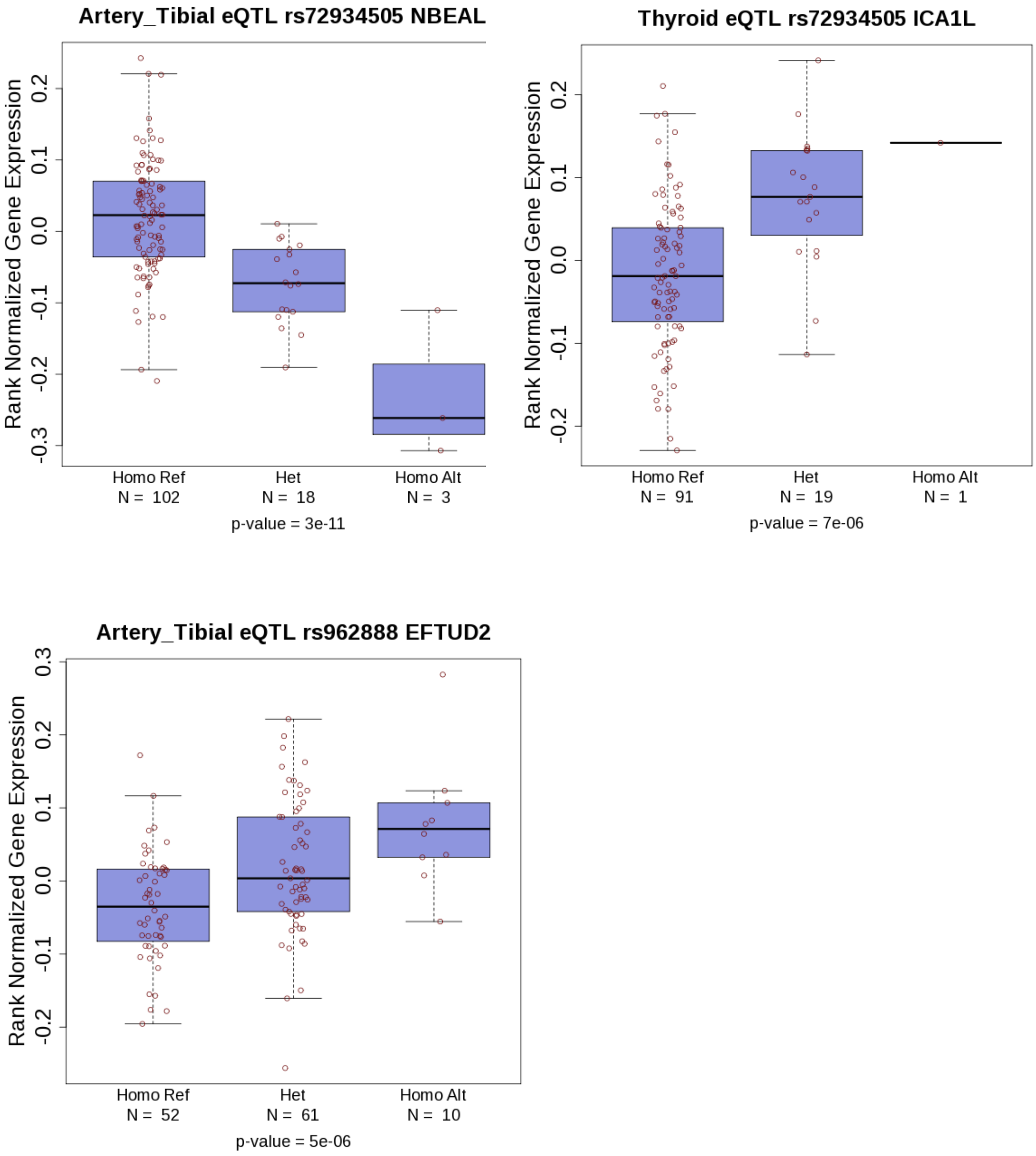

Results are from interrogation of GTEx portal. [7]
