# Supplementary material for: Genome-wide meta-analysis of cerebral white matter hyperintensities in patients with stroke
Source: Neurology. 2016 Jan 12;86(2):146–53. doi: 10.1212/WNL.0000000000002263 (PMC4731688; doi:10.1212/WNL.0000000000002263)
Supplement: Data Supplement [file supp_WNL.0000000000002263_Supplementary_Materials.pdf]

Supplementary Materials

Power Calculation

For a quantitative trait association study with N individuals, the non-centrality parameter (NCP) to detect a causal variant explaining q of the trait variance is given by:[1]

$$NCP = \frac{Nq^2}{1 - q^2}$$

Sampling from a chi-squared distribution with 1 degree of freedom and the above *NCP*, we can calculate power by dividing the number of sampled observations reaching the required significance level ($p<5x10^{-8}$) by the number of sampled observations. The results for our sample of 3,670 individuals are given in Supplementary Figure 1.

Cohort Description

Stroke cases were recruited (with one exception) from hospital-based studies of ischemic stroke patients with genome-wide association study (GWAS) data available as well as MRI scans available for WMH volume measurement. Exclusion criteria were age <18 years, non-European ancestry, monogenetic causes of stroke, vasculitis and any non-ischemic cause of WMH. Cases were assigned a stroke subtype using Trial of Org 10172 in Acute Stroke Treatment (TOAST) classification system by experienced neurologists or stroke specialists.[2]

Australian Stroke Genetics Collaborative (ASGC)

Stroke cases comprised European-ancestry patients admitted to four clinical centers across Australia (The Neurosciences Department at Gosford Hospital, Gosford, New South Wales (NSW); the Neurology Department at John Hunter Hospital, Newcastle, NSW; The Queen Elizabeth Hospital, Adelaide; and the Royal Perth Hospital, Perth) between 2003 and 2008. Stroke was defined by WHO criteria as a sudden focal neurologic deficit of vascular origin, lasting more than 24 hours and confirmed by brain imaging. Other

investigative tests such as electrocardiogram, carotid Doppler and trans-esophageal echocardiogram were conducted to define stroke etiology as clinically appropriate.

Siblings With Ischemic Stroke Study (SWISS)

This is a prospective, multicenter study of sibling pairs with first-ever or recurrent ischemic stroke.[3] Probands were recruited from 70 clinical centers across the US and Canada. Ischemic stroke affected and unaffected siblings were recruited primarily using proband-initiated contact. All affected individuals had WHO-defined stroke confirmed by a study neurologist to be ischemic on the basis of brain imaging. Peripheral blood DNA samples were collected between October 2000 and December 2009.

The Ischemic Stroke Genetics Study (ISGS)

Ischemic Stroke Genetics Study (ISGS) was a 5-center, prospective, case-control study of first-ever ischemic stroke cases. [4] All affected individuals had WHO-defined stroke confirmed by a study neurologist to be ischemic on the basis of head CT or brain MRI. Peripheral blood DNA samples were collected between May 2003 and September 2008.

Massachusetts General Hospital (MGH)

Cases presenting with ischemic stroke and admitted to the Massachusetts General Hospital (MGH) Stroke Unit through the Emergency Department, or evaluated in the MGH Neurology outpatient clinics, as well as on the inpatient Medical and Vascular Surgical services from January 2003 to July 2008. [5] Ischemic stroke was defined as either (1) a radiographically proven (head CT or MRI) infarct associated with the appropriate clinical stroke syndrome, or (2) a fixed neurological deficit persisting more than 24 hours, consistent with a vascular pattern of involvement and without radiographic evidence of demyelinating or other non-vascular disease. All subjects were evaluated by a neurologist upon presentation and clinical and laboratory data were collected during the admission for qualifying ischemic stroke event. All patients had acute brain imaging as well as ancillary diagnostic investigations: cervical and intracranial vessel imaging using CT or MR angiography (75%), cervical ultrasound (24%), echocardiography (86%), and Holter monitoring (16%).

Besta stroke study (Milano)

This study includes consecutive Italian patients referred to Besta Institute from 2000 to 2009 with stroke and included in the Besta Cerebrovascular Diseases Registry (CEDIR).[6] Ischemic stroke cases, first ever or recurrent, confirmed on brain imaging, were selected for this study. An experienced stroke neurologist assessed all cases.

Wellcome Trust Case-Control Consortium 2 (WTCCC2)

The WTCCC2 samples were genotyped as part of the WTCCC 2 ischemic stroke study. Stroke cases were recruited from three centers in the UK (St. George's Oxford and Edinburgh) and one center in Germany, University and Klinikum Großhadern, Ludwig-Maximilians-University, Munich.[6]

WTCCC2-UK: The St George’s Stroke Study consecutively recruited ischemic stroke patients attending cerebrovascular services between 1995 and 2008 (n=1224). The Oxford Vascular Study recruited patients with acute ischemic stroke or transient ischemic attack (TIA) with evidence of infarction on brain imaging between 2002 and 2008 as part of a population-based study (n=896). The Edinburgh Stroke Study prospectively recruited >1200 consecutive stroke inpatients and outpatients between 2002 and 2005. Those with ischaemic stroke, genome-wide data and MRI available and suitable for WMHV measurement were included in this study.

WTCCC2-D: The Munich study recruited consecutively between 2002 and 2008, from a single Stroke Unit with a high rate of MR imaging (>80%) (n=1383). All subjects were over 18 years of age, of self-reported European ancestry and with a diagnosis of ischemic stroke classified according to TOAST by an experienced neurologist or stroke physician. All patients had brain imaging as well as ancillary diagnostic investigations where clinically relevant.

UK Young Lacunar Stroke DNA Study (DNA Lacunar)

DNA Lacunar is a multicentre cohort study, which constitutes a large DNA resource of young patients with well phenotyped lacunar stroke and stroke-free community controls. Between 2005 and 2012, 1,030 white

patients of European ancestry with lacunar stroke, aged ≤ 70 years, were recruited from 72 specialist stroke centres throughout the UK. All patients underwent brain MRI, imaging of the carotid arteries and ECG. Echocardiography was performed when appropriate. All MRI's and clinical histories were reviewed centrally by one experienced stroke physician.

St Georges University of London (SGUL)

This study recruited patients attending cerebrovascular services at St. George's Hospital, London between 2007-2011. All patients had clinically relevant diagnostic workup performed, including brain imaging with magnetic resonance imaging (MRI) as well as ancillary diagnostic investigations including duplex ultrasonography of the carotid and vertebral arteries, echocardiography, Holter monitoring, magnetic resonance angiography (MRA), CT-angiography (CTA) and blood tests

GENESIS

This study recruited patients attending cerebrovascular services at St. George's Hospital, London between 2011-2013. All patients had clinically relevant diagnostic workup performed, including brain imaging with magnetic resonance imaging (MRI) as well as ancillary diagnostic investigations including duplex ultrasonography of the carotid and vertebral arteries, echocardiography, Holter monitoring, magnetic resonance angiography (MRA), CT-angiography (CTA) and blood tests

Leuven Stroke Study

Patients with cerebral ischemia, defined as a clinical stroke with imaging confirmation or a TIA with a new ischemic lesion on diffusion weighted MRI, who were admitted to the Stroke Unit of the University Hospitals in Leuven were enrolled. All patients underwent brain imaging and a standardized protocol including carotid ultrasound or CT angiography and cardiac examination (echocardiography and Holter monitoring) in all patients.

References

1. Yang J, Wray NR, Visscher PM. Comparing apples and oranges: Equating the power of case-control and quantitative trait association studies. *Genet. Epidemiol.* 2010;34:254-257

2. Adams HP, Jr., Bendixen BH, Kappelle LJ, Biller J, Love BB, Gordon DL, et al. Classification of subtype of acute ischemic stroke. Definitions for use in a multicenter clinical trial. Toast. Trial of org 10172 in acute stroke treatment. *Stroke*. 1993;24:35-41

3. Meschia JF, Nalls M, Matarin M, Brott TG, Brown RD, Jr., Hardy J, et al. Siblings with ischemic stroke study: Results of a genome-wide scan for stroke loci. *Stroke*. 2011;42:2726-2732

4. Meschia JF, Brott TG, Brown RD, Jr., Crook RJ, Frankel M, Hardy J, et al. The ischemic stroke genetics study (isgs) protocol. *BMC Neurol*. 2003;3:4

5. Failure to validate association between 12p13 variants and ischemic stroke. *N. Engl. J. Med.* 2010;362:1547-1550

6. Bellenguez C, Bevan S, Gschwendtner A, Spencer CC, Burgess AI, Pirinen M, et al. Genome-wide association study identifies a variant in hdac9 associated with large vessel ischemic stroke. *Nat. Genet.* 2012;44:328-333

7. The genotype-tissue expression (gtex) project. *Nat. Genet.* 2013;45:580-585
