# Supplementary material for: Genome-wide meta-analysis of cerebral white matter hyperintensities in patients with stroke
Source: Neurology. 2016 Jan 12;86(2):146–53. doi: 10.1212/WNL.0000000000002263 (PMC4731688; doi:10.1212/WNL.0000000000002263)
Supplement: Coinvestigators [file supp_86_2_146_v2_index.html]

Coinvestigators 

# Genome-wide meta-analysis of cerebral white matter hyperintensities in patients with stroke

## Coinvestigators

**Neurology® data supplements are not copyedited before publication. Published editorials and translations have been copyedited.  
 © 2016 American Academy of Neurology.  
  
 Files in this Data Supplement:**

- Coinvestigators - Microsoft Word file
