# Supplementary material for: Genome-wide meta-analysis of cerebral white matter hyperintensities in patients with stroke
Source: Neurology. 2016 Jan 12;86(2):146–53. doi: 10.1212/WNL.0000000000002263 (PMC4731688; doi:10.1212/WNL.0000000000002263)
Supplement: Coinvestigators [file supp_WNL.0000000000002263_Coinvestigators.docx]

International Stroke Genetics Consortium Co-investigators:

Sylvia Smoller, PhD (Albert Einstein College of Medicine, Site co-investigator); John Sorkin, MD (Baltimore VA Medical Center, Site co-investigator); Xingwu Wang, MD (Beijing Hypertension League Institute, Site co-investigator); Magdy Selim, MD, PhD (Beth Israel Deaconess Medical Center, Site co-investigator); Aleksandra Pikula, MD, PhD (Boston University Medical Center, Site co-investigator); Philip Wolf, MD, PhD (Boston University School of Medicine, Site co-investigator); Stephanie Debette, MD (Boston University School of Medicine, Site co-investigator); Sudha Seshadri, MD (Boston University School of Medicine, Site co-investigator);

Paul de Bakker, PhD (Brigham and Women's Hospital, Site co-investigator); Daniel Chasman, MD (Brigham and Women's Hospital, Site co-investigator); Kathryn Rexrode, MD (Brigham and Women's Hospital, Harvard Medical School, Site co-investigator); Ida Chen, MD (Cedars Sinai Medical Center, Site co-investigator); Jerome Rotter, MD (Cedars Sinai Medical Center, Site co-investigator); May Luke, MD (Celera, Site co-investigator); Michelle Sale, MD (University of Virginia, Site co-investigator); Tsong-Hai Lee, MD (Chang Gung Memorial Hospital, Linkou Medical Center, Site co-investigator); Ku-Chou Chang, MD (Chang Gung Memorial Hospital; College of Medicine, Chang Gung University, Site co-investigator); Mitchell Elkind, MD, MS (Columbia University, Site co-investigator); Larry Goldstein, MD, PhD (Duke University, Site co-investigator); Michael (Luke) James, MD (Duke University, Site co-investigator); Monique Breteler, MD (Erasmus University, Site co-investigator); Chris O'Donnell, MD (Framingham Heart Study, Site co-investigator); Didier Leys, MD (France, Site co-investigator); Cara Carty, MD (Fred Hutchinson Cancer Research Center, Site co-investigator); Chelsea Kidwell, MD (Georgetown University, Site co-investigator); Jes Olesen, MD (Glostrup Hospital, Site co-investigator); Pankaj Sharma, MD, PhD (Hammersmith Hospitals & Imperial College London, Site co-investigator); Stephen Rich, MD, PhD (University of Virginia Health System, Site co-investigator); Turgot Tatlisumak, MD (Helsinki University Central Hospital, Site co-investigator);

Olli Happola, MD (Helsinki University Central Hospital, Site co-investigator); Philippe Bijlenga, MD (Hôpitaux Universityersitaires de Genève, Site co-investigator); Carolina Soriano, MD (IMIM-Hospital del Mar, Site co-investigator); Eva Giralt, MD (IMIM-Hospital del Mar, Site co-investigator); Jaume Roquer, MD (IMIM-Hospital del Mar , Site co-investigator); Jordi Jimenez-Conde, MD (IMIM-Hospital del Mar , Site co-investigator); Ioana Cotlarcius, MD (Imperial College London, Site co-investigator); John Hardy, MD (Institute of Neurology, UCL, Site co-investigator); Michal Korostynski, MD (Institute of Pharmacology, Krakow, Poland , Site co-investigator); Giorgio Boncoraglio, MD (IRCCS Istituto neurologico Carlo Besta , Site co-investigator); Elena Ballabio, MD (IRCCS Istituto neurologico Carlo Besta , Site co-investigator);

Eugenio Parati, MD (IRCCS Istituto neurologico Carlo Besta , Site co-investigator); Adamski Mateusz, MD (Jagiellonian University, Site co-investigator); Andrzej Urbanik, MD (Jagiellonian University, Site co-investigator); Tomasz Dziedzic, MD (Jagiellonian University, Site co-investigator); Jeremiasz Jagiella, MD (Jagiellonian University, Site co-investigator); Jerzy Gasowski, MD (Jagiellonian University, Site co-investigator); Marcin Wnuk, MD (Jagiellonian University, Site co-investigator); Rafał Olszanecki, MD (Jagiellonian University, Site co-investigator); Joanna Pera, MD (Jagiellonian University, Site co-investigator); Agnieszka Slowik, MD (Jagiellonian University, Site co-investigator); Karol Józef Juchniewicz , MD (Jagiellonian University, Site co-investigator); Christopher Levi, MD (John Hunter Hospital, University of Newcastle, Site co-investigator); Paul Nyquist, MD, PhD (Johns Hopkins School of Medicine, Scientific committee); Iscia Cendes, MD (Joinville Biobank, Site co-investigator); Norberto Cabral, MD (Joinville Biobank, Site co-investigator); Paulo Franca, MD (Joinville Biobank, Site co-investigator); Anderson Goncalves, MD (Joinville Biobank, Site co-investigator); Lina Keller, MD (Karolinska Institutet , Site co-investigator); Milita Crisby, MD (Karolinska Institutet, Sweden, Site co-investigator); Konstantinos Kostulas, MD (Karolinska Institutet; Karolinska University Hospital, Huddinge unit, Site co-investigator); Robin Lemmens, MD (KULeuven, Site co-investigator); Kourosh Ahmadi, MD (London, Site co-investigator); Christian Opherk, MD (Ludwig-Maximilians-Univeritat Munchen , Site co-investigator); Marco Duering, MD (Ludwig-Maximilians-Univeritat Munchen , Site co-investigator); Martin Dichgans, MD (Ludwig-Maximilians-Univeritat Munchen , Site co-investigator); Rainer Malik, PhD (Ludwig-Maximilians-Univeritat Munchen , Site co-investigator); Mariya Gonik, MD (Ludwig-Maximilians-Univeritat Munchen , Site co-investigator); Julie Staals, MD (Maastricht University Medical Centre, Maastricht, the Netherlands, Site co-investigator); Olle Melander, MD, PhD (Malmo University Hospital, Site co-investigator); Philippe Burri, MD (Malmo University Hospital, Site co-investigator); Ariane Sadr-Nabavi, MD (Mashhad University of Medical Sciences, Site co-investigator); Javier Romero, MD, PhD (Massachusetts General Hospital, Site co-investigator); Alessandro Biffi, MD (Massachusetts General Hospital, Site co-investigator); Chris Anderson, MD (Massachusetts General Hospital, Site co-investigator); Guido Falcone, MD (Massachusetts General Hospital, Site co-investigator); Bart Brouwers, MD (Massachusetts General Hospital, Site co-investigator); Jonathan Rosand, MD, MSc (Massachusetts General Hospital, Site co-investigator); Natalia Rost, MD, MSc (Massachusetts General Hospital, Site co-investigator); Rose Du, MD (Massachusetts General Hospital, Site co-investigator); Christina Kourkoulis, BA (Massachusetts General Hospital, Site co-investigator); Thomas Battey, BA (Massachusetts General Hospital, Site co-investigator); Steven Lubitz, MD, PhD (Massachusetts General Hospital, Site co-investigator); Bertram Mueller-Myhsok, MD (Max Planck Institute of Psychiatry, Munich, Site co-investigator); James Meschia, MD (Mayo Clinic, Steering committee); Thomas Brott, MD, PhD (Mayo Clinic, Site co-investigator); Guillaume Pare, MD (McMaster University, Steering committee, Scientific committee); Alexander Pichler, MD (Medical University Graz, Site co-investigator); Christian Enzinger, MD (Medical University Graz, Site co-investigator); Helena Schmidt, MD (Medical University Graz, Site co-investigator); Reinhold Schmidt, MD (Medical University Graz, Site co-investigator); Stephan Seiler, MD (Medical University Graz, Site co-investigator); Susan Blanton, MD (Miami Institute of Human Genomics; University of Miami Miller School of Medicine, Site co-investigator); Yoshiji Yamada, MD (Mie University, Site co-investigator); Anna Bersano, MD (Milan University, Site co-investigator);

Tatjana Rundek, MD (University of Miami, Site co-investigator); Ralph Sacco, MD (University of Miami, Site co-investigator); Yu-Feng Yvonne Chan, MD (Mount Sinai Medical Center, Site co-investigator); Andreas Gschwendtner, MD, PhD (Ludwig-Maximilians-Univeritat Munchen, Site co-investigator); Zhen Deng, MD (Nanfang Hospital, Southern Medical University, Site co-investigator); Taura Barr, MD (National Institutes of Health, Site co-investigator); Katrina Gwinn, MD (National Institutes of Health, Site co-investigator); Roderick Corriveau, MD (National Institutes of Health, Site co-investigator); Andrew Singleton, MD, PhD (National Institutes of Health, Site co-investigator); Salina Waddy, MD (National Institutes of Health, Site co-investigator); Lenore Launer, MD (National Institutes of Health, Site co-investigator); Christopher Chen, MD (National Neuroscience Institute, Singapore General Hospital , Site co-investigator); Kim En Le, MD (National Neuroscience Institute, Singapore General Hospital , Site co-investigator); Wei Ling Lee, MD (National Neuroscience Institute, Singapore General Hospital , Site co-investigator); Eng King Tan, MD (National Neuroscience Institute, Singapore General Hospital , Site co-investigator); Akintomi Olugbodi, MD (Obafemi Awolowo University, Site co-investigator); Peter Rothwell, MD, PhD (Oxford; Radcliffe Infirmary, Site co-investigator); Sabrina Schilling, MD (Paris, France, Site co-investigator); Vincent Mok, MD (Prince of Wales Hospital, The Chinese University of Hong Kong, Site co-investigator); Elena Lebedeva, MD (Russia, Site co-investigator); Christina Jern, MD (Sahlgrenska University Hospital, Scientific committee); Katarina Jood, MD (Sahlgrenska University Hospital, Site co-investigator); Sandra Olsson, MD (Sahlgrenska University Hospital, Site co-investigator); Helen Kim, MD (San Francisco General Hospital; Center for Cerebrovascular Research, Site co-investigator); Chaeyoung Lee, MD (Soongsil University, Site co-investigator); Laura Kilarski, MD (St. George's University of London, Site co-investigator); Hugh Markus, MD (St. George's, University of London, Site co-investigator); Jennifer Peycke, MD (St. George's, University of London, Site co-investigator); Steve Bevan, PhD (St. George's, University of London, Site co-investigator); Wayne Sheu, MD (Taichung Veterans General Hospital, Site co-investigator); Hung Yi Chiou, MD (Taipei Medical University, Site co-investigator); Joseph Chern, MD (Taipei Medical University, Site co-investigator); Elias Giraldo, MD (The University of Tennessee Health Science Center at Memphis, Site co-investigator); Muhammad Taqi, MD (The University of Tennessee Health Science Center at Memphis, Site co-investigator); Vivek Jain, MD (UC Irvine Medical Center, Site co-investigator); Olivia Lam, MD (University of California San Francisco, Site co-investigator); George Howard, MD (University of Alabama School of Public Health, Site co-investigator); Daniel Woo, MD (University of Cincinnati, Steering committee); Steven Kittner, MD (University of Maryland Hospital, Site co-investigator); Braxton Mitchell, PhD, MPH (University of Maryland School of Medicine, Site co-investigator); John Cole, MD (University of Maryland School of Medicine, Site co-investigator); Jeff O'Connell, MD (University of Maryland School of Medicine, Site co-investigator); Dianna Milewicz, MD (University of Texas Medical School at Houston, Site co-investigator); Kachikwu Illoh, MD (University of Texas-Houston, Site co-investigator); Bradford Worrall, MD (University of Virginia Health System, Site co-investigator); Colin Stine, MD (University. of MD School of Medicine, Site co-investigator); Bartosz Karaszewski, MD (University College London, Site co-investigator); David Werring, MD (University College London, Site co-investigator); Reecha Sofat, MD (University College London, Site co-investigator); June Smalley, MD (University College London, Site co-investigator); Arne Lindgren, MD (University Hospital Lund, Steering committee, Scientific committee); Bjorn Hansen, BA (University Hospital Lund, Site co-investigator); Bo Norrving, MD (University Hospital Lund, Site co-investigator); Gustav Smith, MD (University Hospital Lund, Site co-investigator); Juan José Martín, MD (University Hospital Sanatorio Allende; Córdoba, Argentine, Site co-investigator); Vincent Thijs, MD (University Hospitals Leuven, Site co-investigator); Karin Klijn, MD (University Medical Center Utrecht, Site co-investigator); Femke van't Hof, MD, PhD (University Medical Center Utrecht, Site co-investigator); Ale Algra, MD (University Medical Center Utrecht, Site co-investigator); Mary Macleod, MD (University of Aberdeen, Site co-investigator); Rodney Perry, MD (University of Alabama at Birmingham School of Public Health, Site co-investigator); Donna Arnett, MD (University of Alabama at Birmingham School of Public Health, Site co-investigator); Alessandro Pezzini, MD (University of Brescia, Site co-investigator); Alessandro Padovani, MD (University of Brescia, Site co-investigator); Steve Cramer, MD, PhD (University of California Irvine, Site co-investigator); Mark Fisher, MD (University of California Irvine, Site co-investigator); Danish Saleheen, MD (University of Pennsylvania, Site co-investigator); Joseph Broderick, MD (University of Cincinnati, Site co-investigator); Brett Kissela, MD (University of Cincinnati, Site co-investigator);

Alex Doney, MD (University of Dundee, Site co-investigator); Cathie Sudlow, MD (University of Edinburgh; Western General Hospital, Steering committee); Kristiina Rannikmae, MD (University of Edinburgh; Western General Hospital, Site co-investigator); Scott Silliman, MD (University of Florida, Site co-investigator); Caitrin McDonough, MD (University of Florida, Site co-investigator); Matthew Walters, MD (University of Glasgow, Site co-investigator); Annie Pedersen, MD (University of Gothenburg, Site co-investigator); Kazuma Nakagawa, MD (University of Hawaii, Site co-investigator); Christy Chang, MD (University of Maryland, Site co-investigator); Mark Dobbins, MD (University of Maryland,, Site co-investigator); Patrick McArdle, PhD (University of Maryland, Site co-investigator); Yu-Ching Chang, MD (University of Maryland, Site co-investigator); Robert Brown, MD (University of Michigan, Site co-investigator);

Devin Brown, MD (University of Michigan, Site co-investigator); Elizabeth Holliday, MD (University of Newcastle, Site co-investigator); Raj Kalaria, MD (University of Newcastle, Site co-investigator); Jane Maguire, MD (University of Newcastle; John Hunter Hospital, Steering committee); John Attia, MD (University of Newcastle; John Hunter Hospital, Site co-investigator); Martin Farrall, MD (University of Oxford; Wellcome Trust Center for Human Genetics, Site co-investigator); Anne-Katrin Giese, MD (University of Rostock, Germany, Site co-investigator); Myriam Fornage, MD (University of Texas- Houston; Health Sciences Center, Scientific committee); Jennifer Majersik, MD (University of Utah, Scientific committee); Mary Cushman, MD (University of Vermont and Fletcher Allen Health Care, Site co-investigator); Keith Keene, MD (University of Virginia, USA, Site co-investigator); Siiri Bennett, MD (University of Washington, Site co-investigator); David Tirschwell, MD, MSc (University of Washington, Site co-investigator); Bruce Psaty, MD (University of Washington, USA, Site co-investigator); Alex Reiner, MD (University of Washington, USA, Site co-investigator); Will Longstreth, MD (University of Washington; Harborview Medical Center, Site co-investigator); David Spence, MD (University of Western Ontario, Robarts Research Institute, Site co-investigator); Joan Montaner, MD (Vall d’Hebron Hospital, Site co-investigator); Israel Fernandez-Cadenas, MD (Vall d’Hebron Hospital, Steering committee); Carl Langefeld, MD (Wake Forest University, Site co-investigator); Cheryl Bushnell, MD (Wake Forest University Health Sciences, Site co-investigator); Laura Heitsch, MD (Washington University of St. Louis, Site co-investigator); Jin-Moo Lee, MD, PhD (Washington University of St. Louis, Site co-investigator); Kevin Sheth, MD (Yale New Haven Hospital, Yale School of Medicine, Site co-investigator)
